# Development of a Microplate Platform for High-Throughput Sample Preparation Based on Microwave Metasurfaces

**DOI:** 10.1109/access.2021.3063092

**Published:** 2021-03-08

**Authors:** ZACH E. NICHOLS, LAHARI SAHA, RACHAEL KNOBLAUCH, TONYA M. SANTAUS, CHRIS D. GEDDES

**Affiliations:** 1 Department of Chemistry and Biochemistry, University of Maryland, Baltimore County, Catonsville, MD 21250, USA; 2 Institute of Fluorescence, Baltimore, MD 21202, USA

**Keywords:** Biochemical analysis, biomolecule fragmentation, clinical diagnosis, microwave ovens, microwave metasurfaces, sample preparation

## Abstract

Sample preparation is one of the most time-consuming steps in diagnostic assays, particularly those involving biological samples. In this paper we report the results of finite-difference time-domain (FDTD) simulations and thermographic imaging experiments carried out with the intent of designing a microplate for rapid, high-throughput sample preparation to aid diagnostic assays. This work is based on devices known as microwave lysing triangles (MLTs) that have been proven capable of rapid sample preparation when irradiated in a standard microwave cavity. FDTD software was used to model a microplate platform as a polystyrene substrate with an array of various passive scattering elements (PSEs) of different sizes, shapes, and interelement spacings in a 2.45 GHz field identical to that of a common microwave oven. Based on the FDTD modeling, several PSE arrays were fabricated by cutting PSEs out of aluminum foil and adhering them to the bottom of regular polystyrene microplates to make prototypes. Each prototype microplate was then irradiated in a microwave cavity for a range of different times, powers, and source angles and the heating effects were observed via a forward looking infrared (FLIR) camera. Based on the results, two prototype microplate platforms have been shown to demonstrate electromagnetic and thermal enhancements similar to those seen with MLTs as well as tunable thermal responses to radio frequency (RF) irradiation.

## I. INTRODUCTION

**FIGURE 1.**
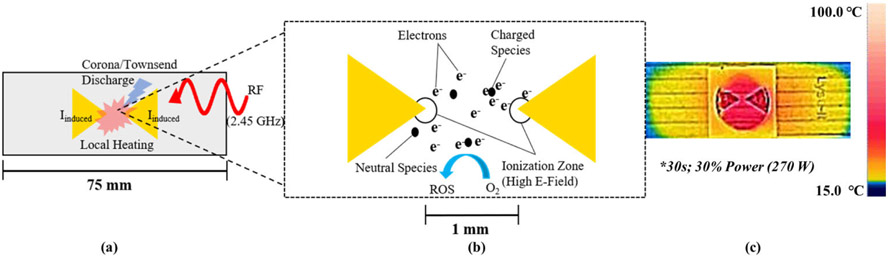
(a) Microwave lysing triangles (MLTs) during irradiation. (b) Depiction of the electric phenomenon that occur upon irradiation with radio frequency (RF) radiation including the generation of reactive oxygen species (ROS). (c) Thermal image showing the distribution of heat after RF irradiation in a microwave oven.

**FIGURE 2.**
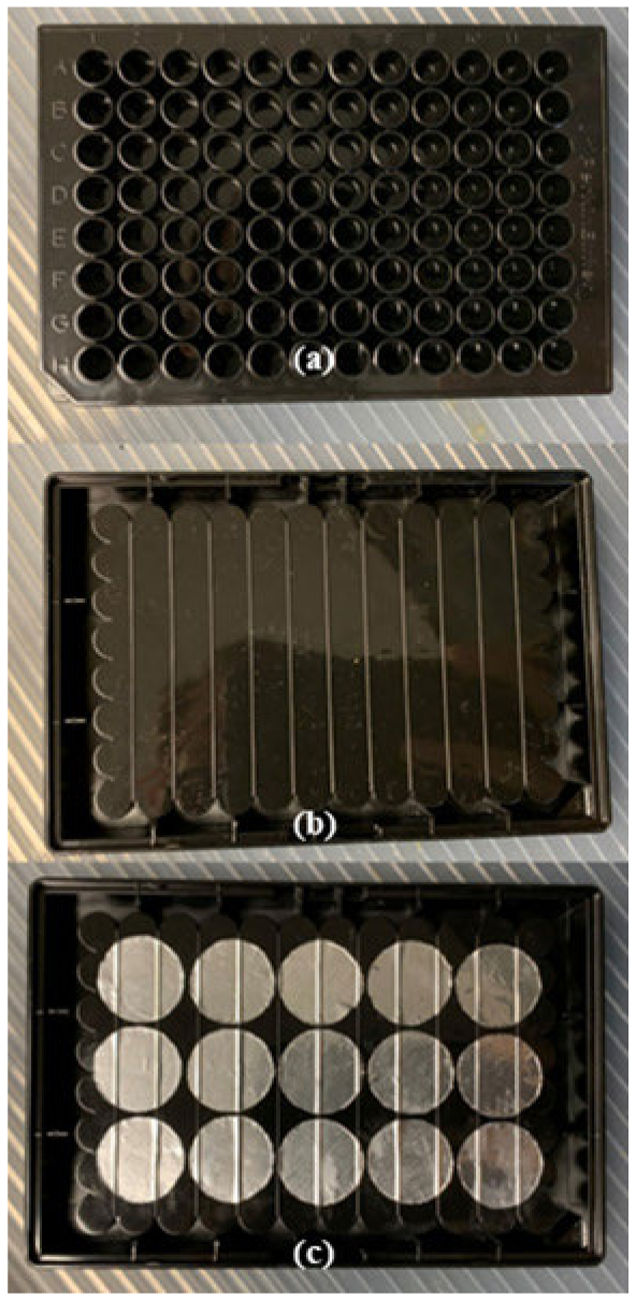
(a) Top view and underside views of a 96-well microplate (b) without and (c) with arrays of passive scattering elements (PSEs).

Sample preparation is an essential process in many laboratories but is also often the main bottleneck in turnaround time as well as the primary source of error and cause of discrepancies between laboratories in many analytical schemes [1]-[3]. In addition, the method of sample preparation used significantly affects the quality of results, the sample throughput, and the cost of a given testing method [2]. One of most important applications of sample preparation in a modern laboratory is in the diagnosis of infectious diseases, as they are one of the leading causes of morbidity and mortality globally [4]. Rapid and accurate diagnosis is critical for identification and treatment as well as in monitoring outbreaks of disease [4]-[7]. Besides increasing time and costs, sample preparation constraints also limit diagnosis in point-of-care (POC) and resource-limited settings due to human samples, such as blood or oral fluids, often being in complex sample matrices [4]-[8], [10]. One of the devices that has been developed to reduce preparation time and cost are microwave lysing triangles (MLTs). MLTs consist of two gold, equilateral triangles roughly 12.3 mm in height, oriented vertex-to-vertex on a glass substrate approximately the size of a microscope slide. When irradiated in a microwave cavity, MLTs focus the electromagnetic field intensity between the vertices of the triangles, which leads to rapid heating of the sample, extraction of proteins or nucleic acids, fragmentation of nucleic acids, deactivation of enzymes such as RNases and DNases, and generation of reactive oxygen species (ROS) on timescales ranging from 30-120 seconds [11]-[13]. In addition to this, MLTs have proven capable of rapid sample preparation for various pathogens including *E. coli, Listeria monocytogenes*, and *Vibrio cholerae*, and are better suited for low-resource settings and cold chain transport compared to traditional methods [13], [14]. The rapid sample preparation abilities of MLTs arise from both their thermal effects and their ability to focus the microwave-frequency electromagnetic fields generated by a common microwave oven. From classical electrodynamics, it is known that a time-varying electromagnetic field (2.45 GHz) generated by the microwave oven induces current in the planar metal triangles. Depending on the power of the microwave source and the irradiation time this leads to heating and occasionally corona or Townsend discharge between the triangles, shown in Fig. 1(a). Based on FDTD simulations and the detection of ROS in samples treated with MLTs, it has been shown that the region between the vertices of the triangles creates a region of high electric field intensity, as shown in Fig. 1(b). This region of high intensity in turn leads to ionization of molecules in the sample, such as oxygen, that are within range of the focused E-field [13], [19], [20]. Rapid heating of samples also occurs, as shown in Fig. 1(c), and is capable of thermally lysing bacterial pathogens [13], [19]. Despite these advantages, the main drawback of MLTs is that their sample throughput is limited to one sample per MLT [14]. One way of overcoming this limitation is to develop a 96-well microplate that allows for 96 individual samples to be prepared simultaneously, as similar analytical techniques have done [16]-[18], [20]. This would also increase sample throughput due to microplate compatibility with existing laboratory equipment and ease of automation [15]-[18]. In this study, we pursued this idea in order to develop a microplate platform for high-throughput sample preparation with the electromagnetic and thermal effects of MLTs. Using finite-difference time-domain (FDTD) simulations to observe how different arrays of passive scattering elements (PSEs) on the surface of a substrate focused electric field intensity in a 2.45 GHz field, several potential arrays of PSEs were identified. After determining which arrays showed the greatest electric field enhancements in FDTD simulations, a prototype microplate was made by adhering passive scattering elements (PSEs) nearly identical to those in the simulation to an actual 96-well microplate, as shown in Fig. 2., and observing its thermal effects upon irradiation.

## II. EXPERIMENTAL

### A. FINITE-DIFFERENCE TIME-DOMAIN (FDTD) SIMULATIONS

**FIGURE 3.**
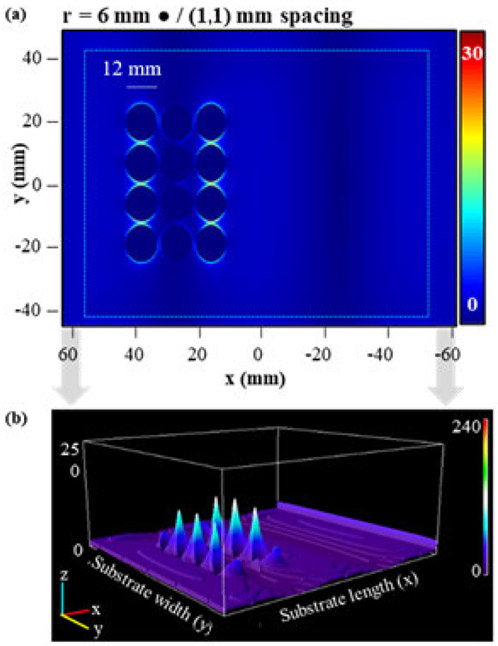
(a) 2D contour plot obtained from a half-covered substrate FDTD simulation showing electric field amplitude, Re(E) (V/m) on the substrate with an array with disk-shaped PSEs of 6 mm radius and 1 mm interelement spacings in the X and Y directions. (b) a 3D representation of the electric field magnitude from (a) with magnitude transposed to the z-axis.

**FIGURE 4.**
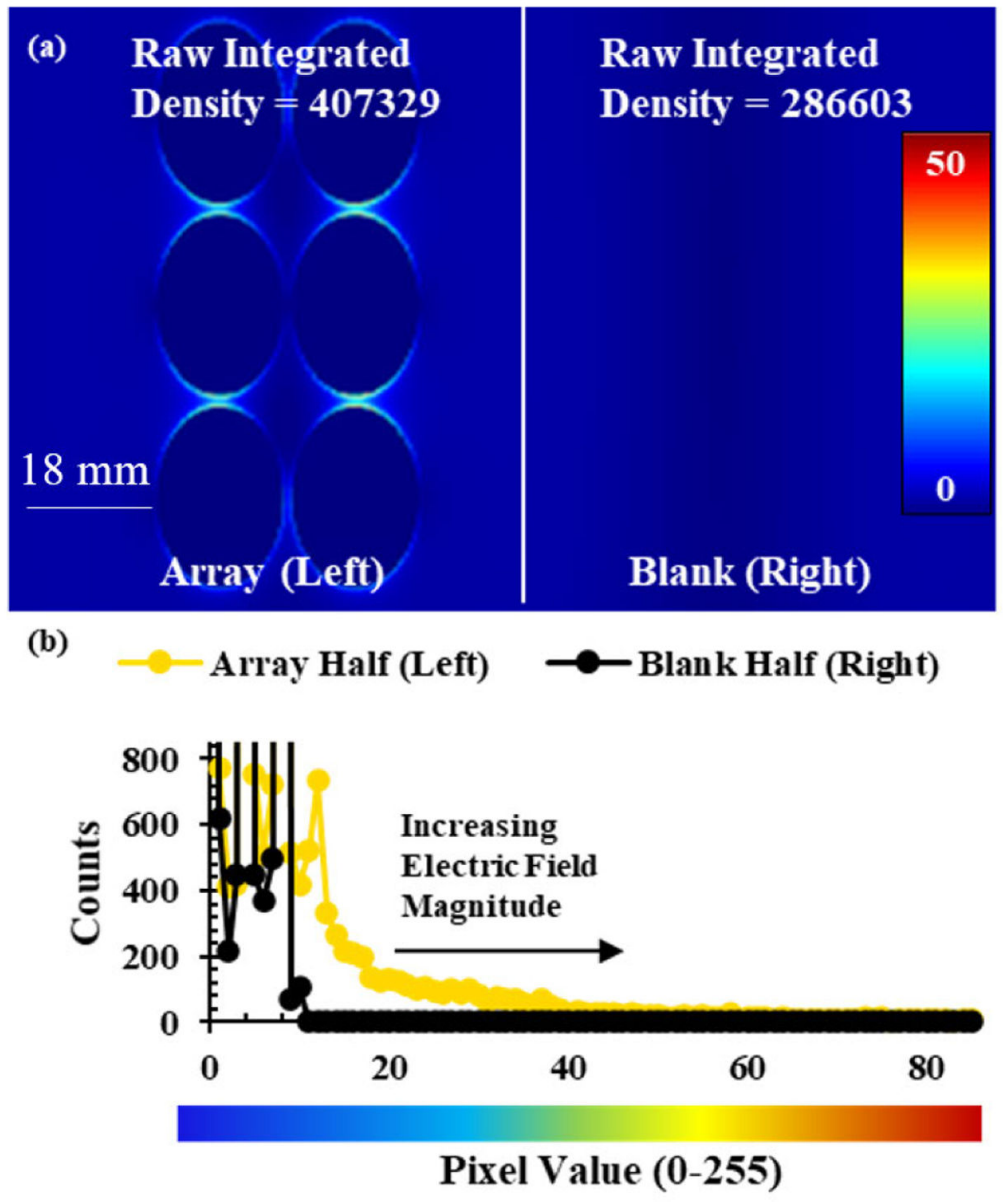
(a) 2D contour plot of the polystyrene substrate and metal arrays from a half-covered substrate FDTD simulation with gold, disk-shaped PSEs of 9 mm radius and 1 mm X and Y interelement spacing on the left half and a blank right half. (b) Graphical representation of each half of the image in greyscale pixel values (0-255).

All FDTD simulations were performed on a Dell T-3600 workstation (4 cores) using Lumerical FDTD solver software (Ansys). Each simulation used a 150 mm square and 100 mm high mesh with dx = 0.1 mm and dy = 0.1 mm. To model microwave irradiation two plane wave sources (Bloch/Periodic type, 2.45 GHz frequency, 0.545963 GHz bandwidth, 0.808244 ns pulse length, 2. 29163 ns offset, amplitude of 1) were placed 150 mm from the center of a 3D polystyrene rectangle, (dimensions: 127.76 mm by 85.47 mm by 4 mm, 1.59 index of refraction) representing the substrate, facing each other to simulate the standing wave that is created in a microwave cavity. The boundary conditions for each simulation were set as stretched coordinate perfectly matched layers (PML) for the maximums and minimums in each dimension. The PML settings for the boundary conditions were standard profile, 8 layers, a kappa value of 2, a sigma value of 1, third order polynomial, an alpha value of 0, a first order alpha polynomial, minimum of 8 layers, and maximum of 64 layers. All PSEs used in the simulations were of thickness 0.0001 mm and the optical constants used were the CRC presets in the software. Electric field measurements were collected with a frequency-domain field and power density functional theory (DFT) monitor object, with a 2D monitor for measuring E-field intensity, ∣**E**∣^**2**^, at specific heights and a 3D monitor for all other simulations. For a visual representation of the FDTD setup see Fig. S1. Electric field data were compared by converting 2D-contour plots of simulation results, shown in Fig. 3(a), into 16-bit greyscale and using an image processing program (ImageJ) to create a distribution of pixel values that correspond to the E-field values. Half-covered substrate simulations were used to determine E-field enhancements by simulating a substrate with an array on only one half and the other half being left empty. The E-field intensity of each half of the substrate, shown in Fig. 4(a), was compared using raw integrated density (RawIntDen), which is a summation of all pixel values in a defined area. The ratio of the half with a PSE array (L) and the blank half (R), L/R, was used to quantify E-field enhancement due to the presence of the array, where L and R are the RawIntDen values of the array and blank halves of the substrate. This ratio was converted into a percent, % L/R, to describe the degree of E-field enhancement attributed to the PSE array. Histograms of the range of pixel values were also generated to compare the dispersity of the electric field, using the dispersity index (DI), for different simulations as shown in Fig. 4(b). Selected PSE arrays were then expanded to cover the full substrate and were compared to a blank substrate to observe E-field enhancement with fully covered FDTD simulations. Fully covered substrate simulations using aluminum PSEs were used to study the effects of RF source orientation and distance from the array on the electromagnetic field effects. showing the additive effects of the array on electric field magnitude. Changes in orientation of the RF source were simulated by rotating the plane wave sources used to simulate microwave irradiation in the FDTD simulation by 55 and 90 degrees about the Z-axis relative to their original position (0 degrees). The effects of distance above the array were simulated using 2D power and field monitor objects with dimensions equal to those of the 3D monitor with heights ranging from 0-12mm above the array of PSEs in 2 mm increments.

### B. THERMOGRAPHY

Microwave irradiation was performed using a microwave oven (Frigidaire, Model No. FFCM0934LB, 900 W rated output power, 2450 MHz frequency) with internal dimensions of 31.4325 cm × 34.29 cm × 22.225 cm (Width × Depth × Height). The roller ring was removed from the microwave oven to make the turntable stationary during irradiation except during rotating orientation experiments. All microplates used were OptiPlateTM-96 F model plates (Perkin Elmer) with dimensions of 127.76 cm × 85.47 cm × 14.60 cm (L × W × H). All 96 wells were filled halfway (200 *μ*L) with deionized water before irradiation except where noted. Thermal images were taken immediately after irradiation using a FLIR ONE (FLIR Systems) camera attachment for smartphones on a tripod. Analysis was performed according to the manufacturer’s directions using FLIR Tools software package (FLIR Systems) and temperatures for each microplate were determined by a box of equal area (275 × 180 pixels) for each image. Each microplate underwent 5 trials for 30, 60, and 90 s irradiation times at full (900 W) microwave power as well as 60 s of irradiation at 30% (270 W), 50% (450 W), and 70% (630 W) microwave power. The same microplate platform was used for each trial unless damage during irradiation was observed. These were repeated for both the horizontal and vertical orientations on a stationary turntable and on a rotating turntable for the rotating orientation. Greyscale thermal images of each trial were converted into histograms using ImageJ to characterize breadth of heating using full width at half-max (FWHM), similar to the dispersity index for FDTD images. Thermal images from each individual trial were used to obtain corresponding histograms for each microplate, which were then averaged together to obtain the final distribution for each trial. Trials were run for a blank microplate, a microplate with a 3 × 5 array (9 mm radius disks with 1 mm X and Y interelement spacing), and a 3 × 6 array (13.856 mm sided rhombi with 3 mm X and 1 mm Y interelement spacing).

## III. RESULTS AND DISCUSSION

### A. FINITE-DIFFERENCE TIME-DOMAIN (FDTD)

**FIGURE 5.**
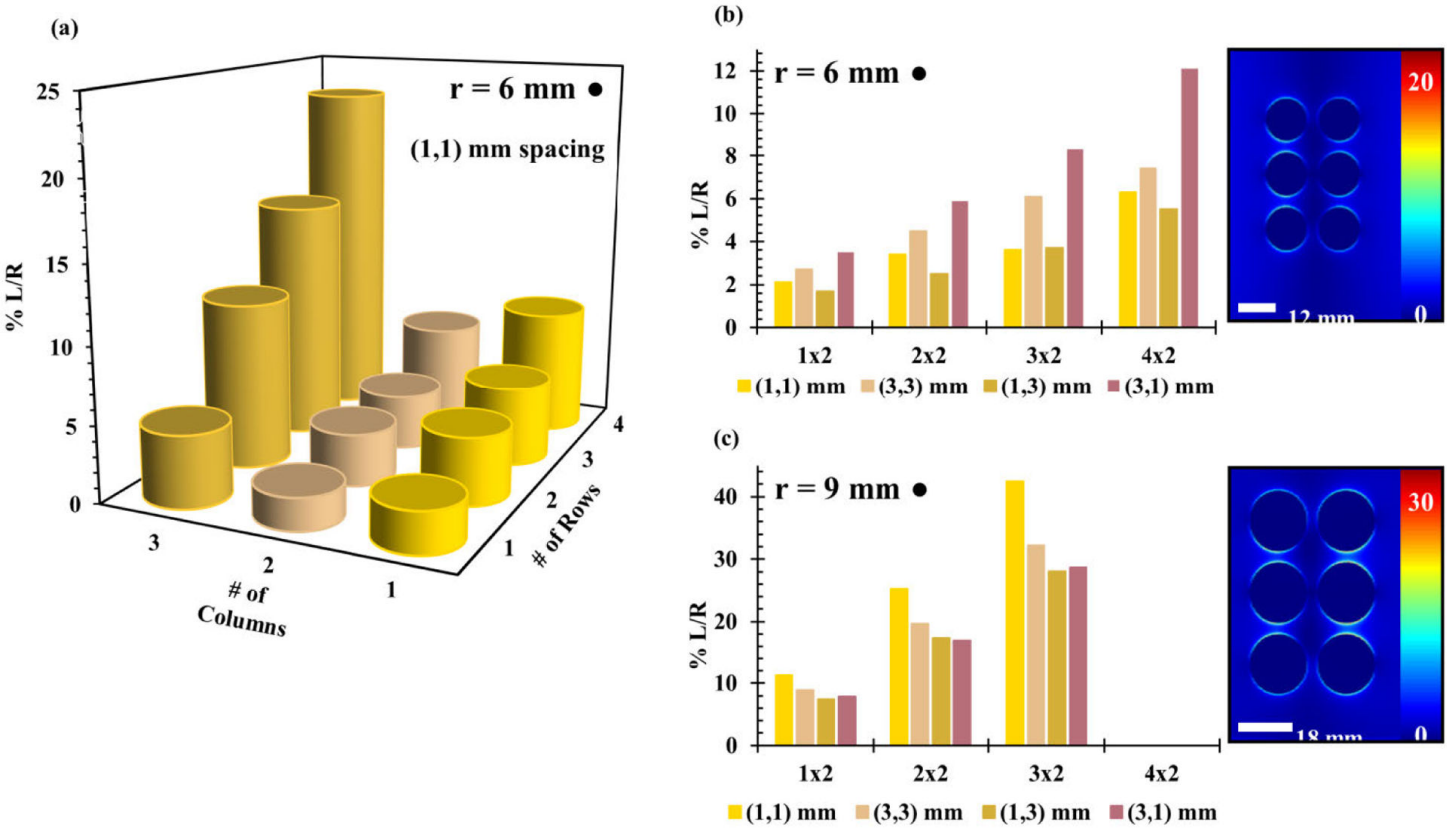
(a) Percent electric field magnitude (∣E∣) increase for half-covered substrate FDTD simulations (% L/R) as a function of rows and columns for a half-covered substrate simulation of gold disks with radius 6 mm and 1 mm interelement X and Y spacing. Comparisons of % L/R electric field magnitude increase for disks of (b) 6 mm and (c) 9 mm radii and different X and Y interelement spacings, noted in the legends.

**FIGURE 6.**
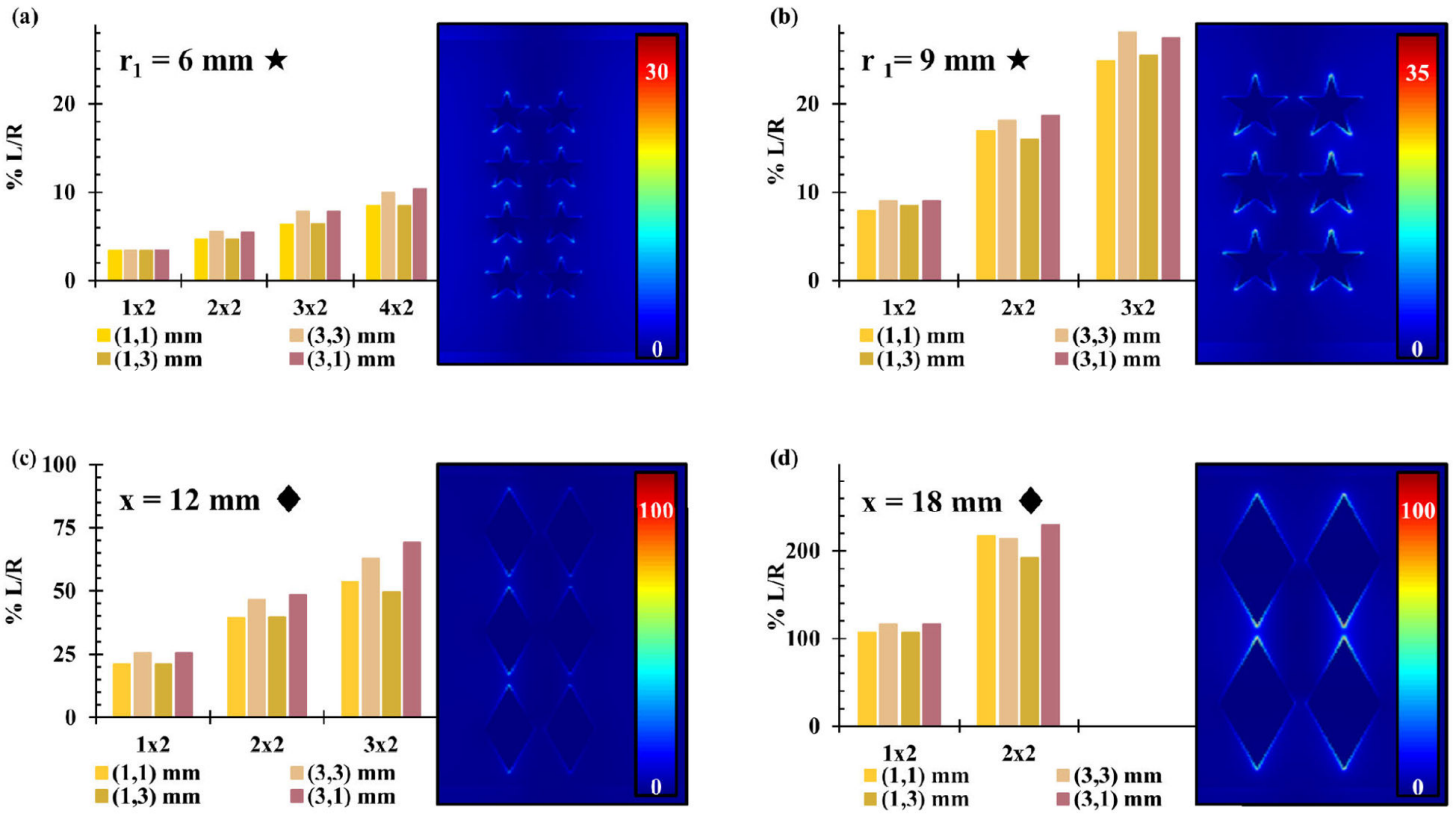
Comparison of the electric field amplitude in terms of %L/R for half-covered substrate FDTD simulations of (a) 9 mm and (b) 6 mm radius star and (c) 12 mm and (d) 18 mm side rhombi arrays with different interelement spacings.

Several different arrays of gold PSEs of different shapes, shown in Fig. S2, were simulated using half-covered substrate FDTD simulations. Half-covered substrates were initially used to save on computing time and resources while still measuring the PSE effects on the E-field. The initial sizes of the PSEs were chosen to be approximately equal to 12 mm in size or approximately (λ/10) for a 2.45 GHz field, and then increased proportionally. This was first conducted with disk-shaped PSEs of radii 6 mm and 9 mm, shown in Fig. 5(a/b) and Fig. 5(c) respectively, due to their simplicity and similarity to spheres. These data demonstrate that the scattering element size (Fig. 5(a) and (b)) and larger PSEs (r = 9 mm) showed greater field enhancements (Fig. 5(c)) than their smaller counterparts (r = 6 mm). Generally, the electric field enhancement increases as the number of rows and columns in an array increase and interelement spacing remains constant. Based on the results observed with disk-shaped PSEs, the analogous versions with 5-pointed stars were chosen to observe if PSEs with sharper points would lead to greater E-field intensity. Data for various arrays, interelement spacings, and element sizes are shown in Fig. 6(a) and (b). When it was observed that they exhibited weaker electric field effects than the disk-shaped PSEs, the analogous rhombi-shaped PSEs were also simulated because of their similarity in shape to the triangles used for MLTs. The results, shown in Fig. 6(c) and (d), demonstrate that the arrays with larger rhombi-shaped PSEs showed similar trends to the disk-shaped PSEs, that is PSEs with larger sizes showed greater field enhancements while interelement spacings had a significant effect on the electric field. These arrays most notably showed that rhombi-shaped PSEs had much greater field enhancements than those seen with disk-shaped PSEs and that 5-pointed star-shaped PSEs showed weaker field enhancements than either.

**FIGURE 7.**
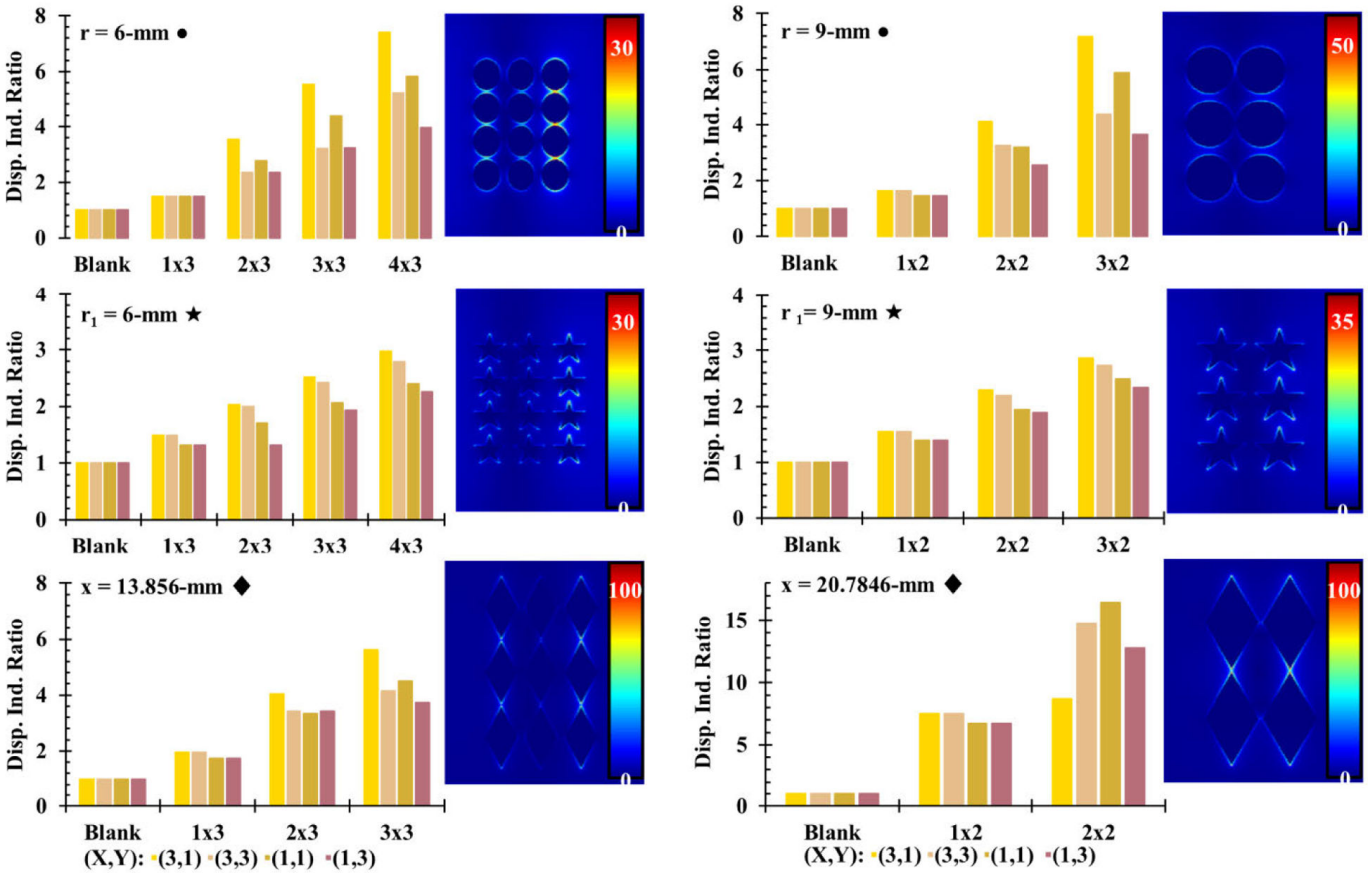
Comparison of the dispersity indices of the electric field magnitude for half-covered substrate FDTD simulations with disks (•), stars (★), and rhombi (♦) arrays with different PSE sizes and spacings.

**FIGURE 8.**
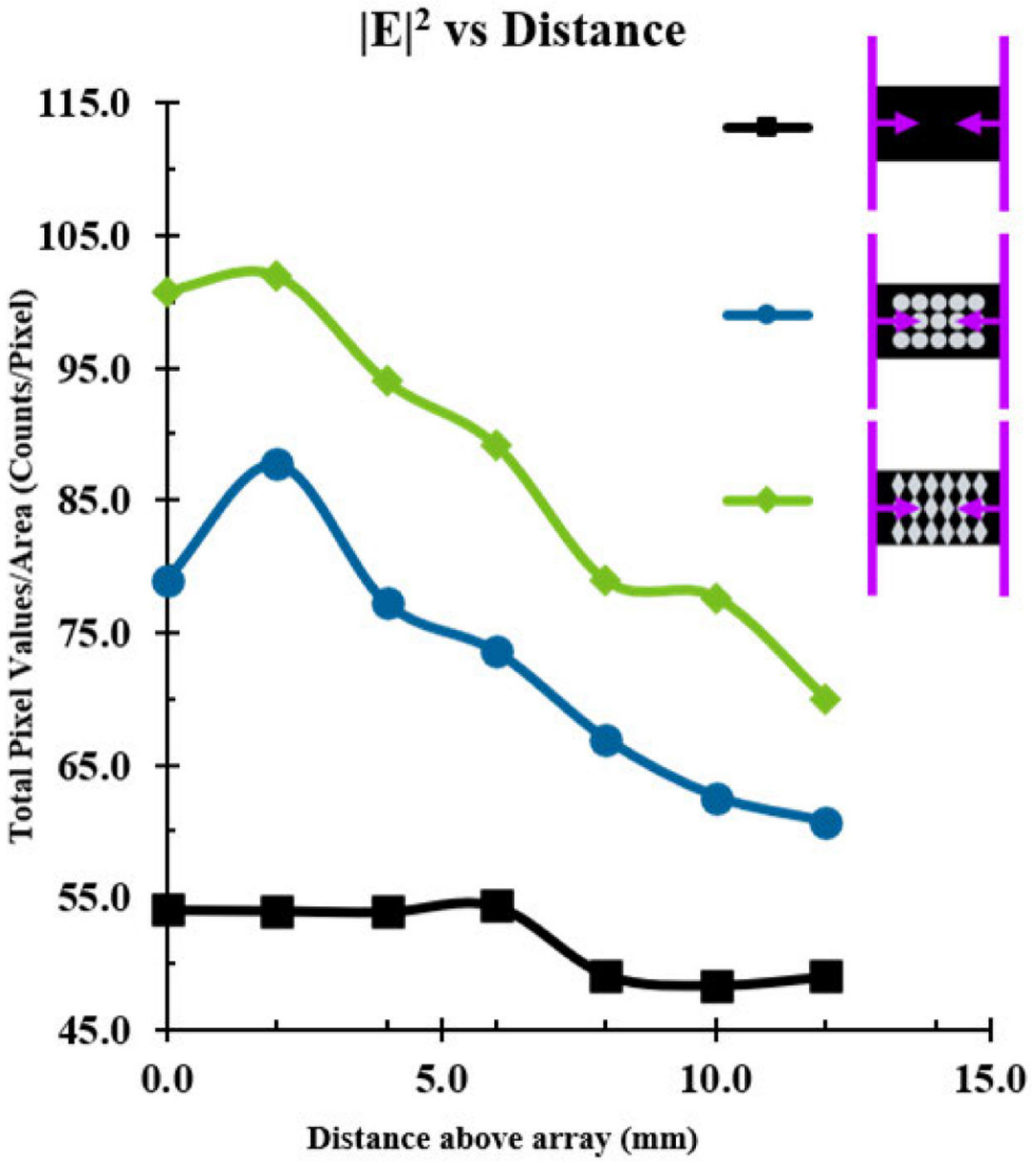
Graph of electric field intensity ∣E∣^2^ as a function of distance from the bottom of the microplate for blank substrate (Black), a 3 × 5 array (9 mm radius disks with 1 mm X and Y spacing, Blue), and a 3 × 6 array (13.856 mm side rhombi with 3 mm X spacing and 1 mm Y spacing, Green). All RF sources were oriented horizontally in the simulations.

In addition to the overall E-field enhancement, the dispersity of E-field intensity was characterized by the ratio of the DI of each half-covered substrate simulation to the DI of a completely blank substrate simulation (DI Ratio = DI_array_ / DI_blank_) to determine how concentrated electric field intensity was in the area of the substrate with an array compared to the area with no array (Fig.7). While the trends in dispersity followed those that were observed for E-field intensity with 6 mm radius disks, 6 mm radius 5-pointed stars, and 13.856 mm sided rhombi arrays, they also showed an increase with the number of rows in each array regardless of interelement spacings. Notably, the greatest electric field dispersity for each array was with interelement spacings of 3 mm in the X direction and 1 mm in the Y direction with the exception of the rhombi-shaped PSEs with 20.7846 mm sides which showed the greatest dispersity with 1 mm interelement spacing in both the X and Y directions. This appears to show that the interelement spacing of the PSEs has a greater effect on electric field dispersity than the shape of the PSEs. Based on the results of the half-covered substrate simulations, several PSE arrays were identified that maximize the E-field enhancements. The optimal interelement spacings for disk and rhombi-shaped PSEs were determined to be: 1 mm X and Y spacing for 9 mm radius disks, 3 mm X and 1 mm Y spacing for 6 mm radius disks, and 3 mm X and 1 mm Y spacing for rhombi of both 13.856 mm and 20.7846 mm sides. Arrays of star-shaped PSEs were not pursued further due to their poor E-field enhancements. Once these arrays were selected, fully covered substrate simulations were carried out for better comparison to the subsequently crafted prototype microplates. In order to make prototype microplates, aluminum foil was determined to be sufficient for creating mock-ups of PSEs before using gold. To verify this, key simulations were repeated with aluminum PSEs to determine if there would be a significant deviation from gold PSEs, the comparison of which can be seen in Fig. S3 for two different arrays of disk-shaped PSEs. Based on those results, there did not seem to be considerable differences between Al and Au PSEs in terms of E-field enhancement, since identical arrays showed the similar results with only 0-1 % L/R difference for disk-shaped PSEs with 6 mm radii and 5-10 % L/R difference for disk-shaped PSEs with 9 mm radii, see Fig. S3. Following the determination of the best arrays and the suitability of aluminum PSEs, simulations of fully covered substrates using aluminum were carried out to better characterize the electric field effects and observe what E-field profiles would look like for an actual microplate platform with a full array. Additionally, new simulations were carried out to observe how electric field intensity varies with distance from the arrays and the effect of source orientation. MLTs have shown ionizing capabilities that vary with these parameters, which an equivalent microplate platform would need to be able to reproduce. The orientation effects were also tested to determine the optimal orientation for a microplate in a microwave oven for sufficient electric field strength and distribution. Since samples in the wells of a 96-well microplate are further from the array of PSEs than samples in MLTs are, the intensity of the electric field at the equivalent height in a microplate needs to be strong enough to ensure ionization. For reference, in a physical 96-well microplate the distance of the headspace from the array is approximately 4 mm with 200 *μ*L of solution in the well. The data shown in Fig. 8 demonstrate the distance dependence of electric field intensity for a blank substrate and two of the fully covered substrate simulations, which both appear to peak around 2 mm above the array, and decay exponentially with distance thereafter. This is not entirely unexpected as many related physical phenomena obey inverse-square laws, such as Coulomb’s law in electrostatics and the radiant intensity of light from a point source. The trend in electric field intensity observed here could be expected to obey an inverse-square law similarly due to the array acting as a transmitter or reflector of the field in the microwave. Because of these results, it is likely that the electric field intensity experienced by the headspace of liquid in a microplate is sufficient other charged species that have enough to generate the reactive oxygen species (ROS) and been previously observed with MLTs [13]. The 2D contour images at varying heights for this array can be seen in Fig. S4 for the array with disk-shaped PSEs. The same data for the vertical and 55-degree rotated orientations of the disk array at distances 0-12 mm above the array can be seen in Fig. S5 and Fig. S6. The orientation experiments show that each array appeared to have lower overall electric field intensity in the vertical orientation than it did in the horizontal orientation regardless of the array. While the 55-degree rotated simulations do show greater intensity than the vertical orientation, they are still not as robust as the simulations in the horizontal position. Based on these results, it was expected that the actual microplate prototypes would show similar relationships to orientation in a microwave oven.

### B. THERMOGRAPHY AND MICROWAVE HEATING

**FIGURE 9.**
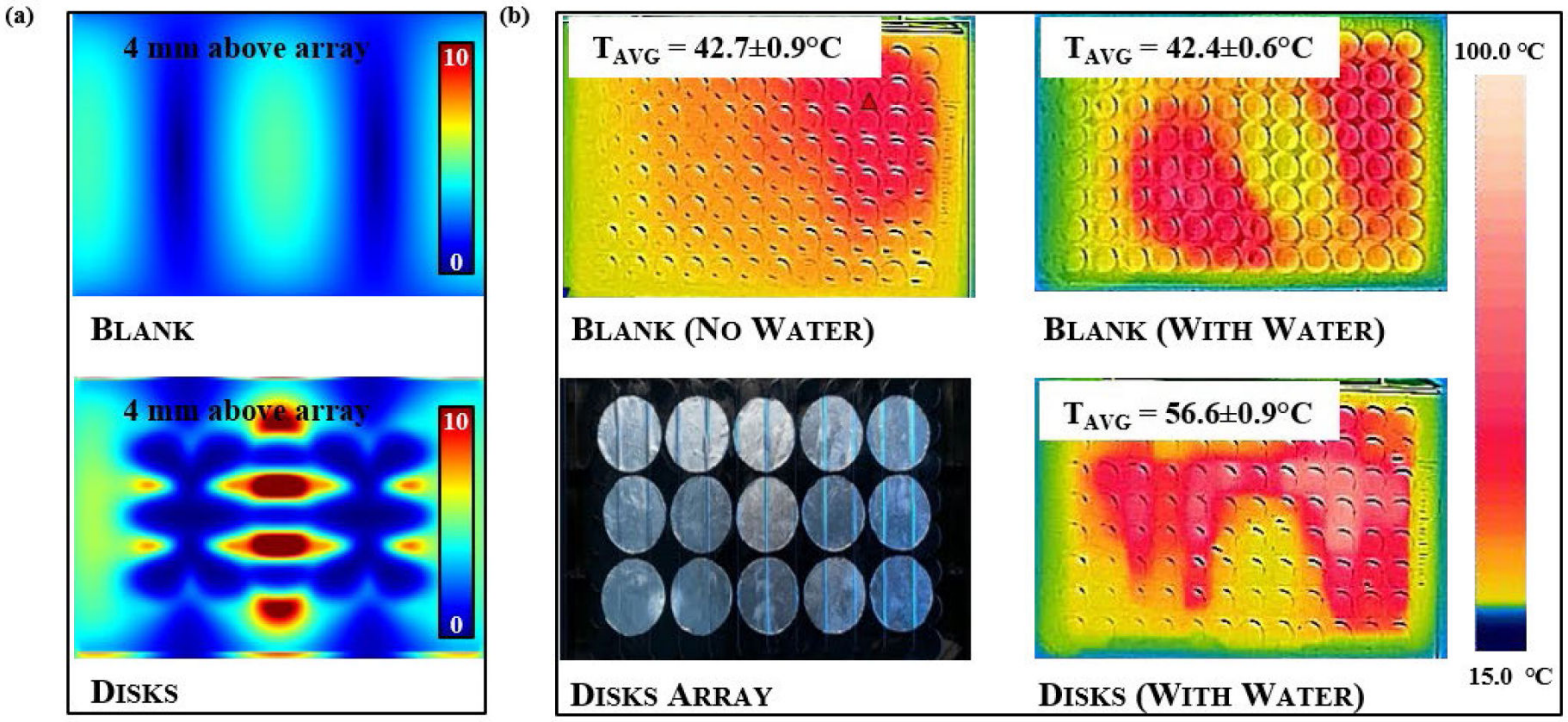
(a) Comparison of electric field intensity 4 mm above the array and (b) thermal patterns after heating at 90s; 100% power (900 W) for a regular 96-well microplate (top row) and a 96-well microplate with 9 mm radius disks (bottom row).

**FIGURE 10.**
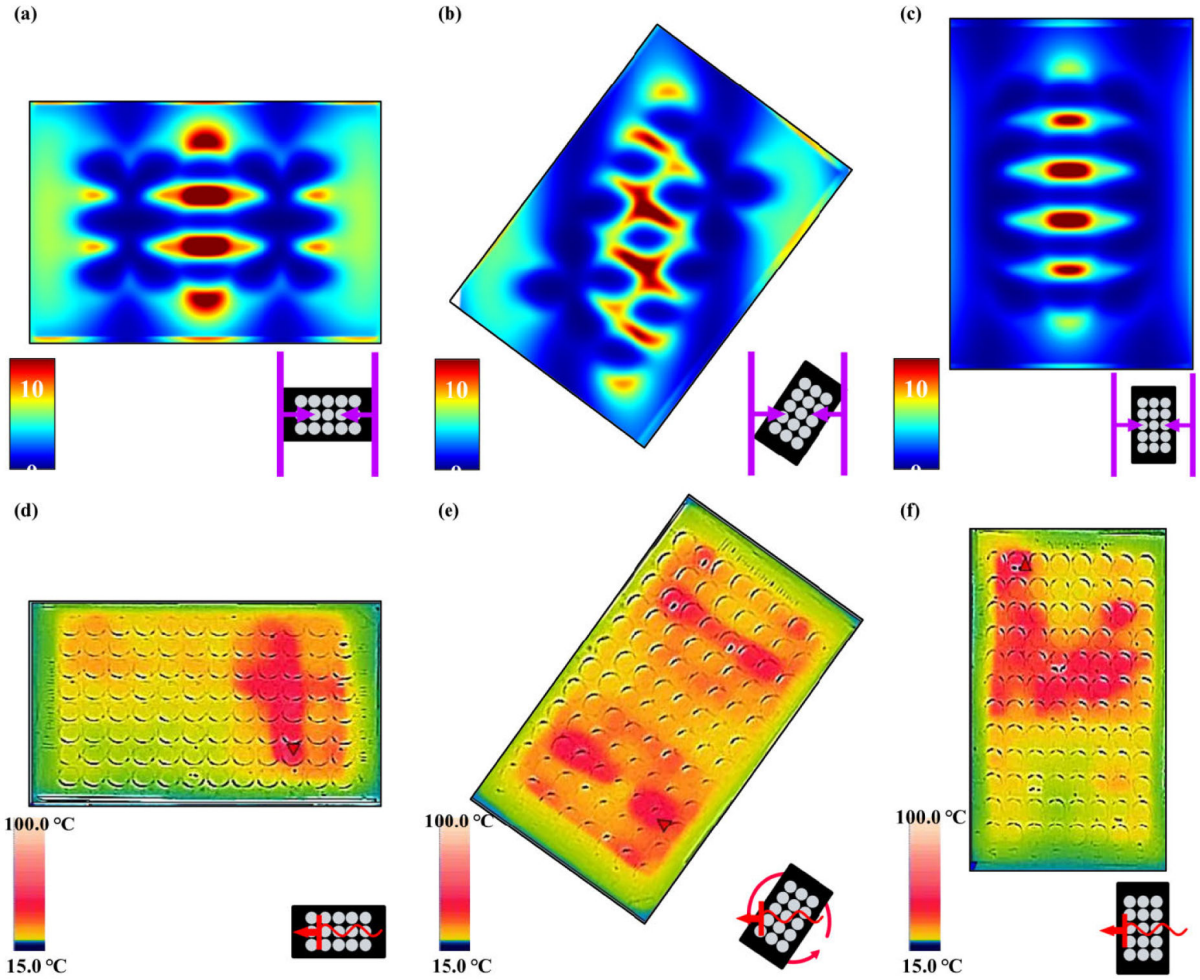
Comparison of electric field intensity (∣E∣^2^) profiles 4 mm above the array (a-c) and thermal data for different orientations of arrays with 9 mm radius disk-shaped PSEs after heating at 30s; 100% Power (900 W) (d-f).

**FIGURE 11.**
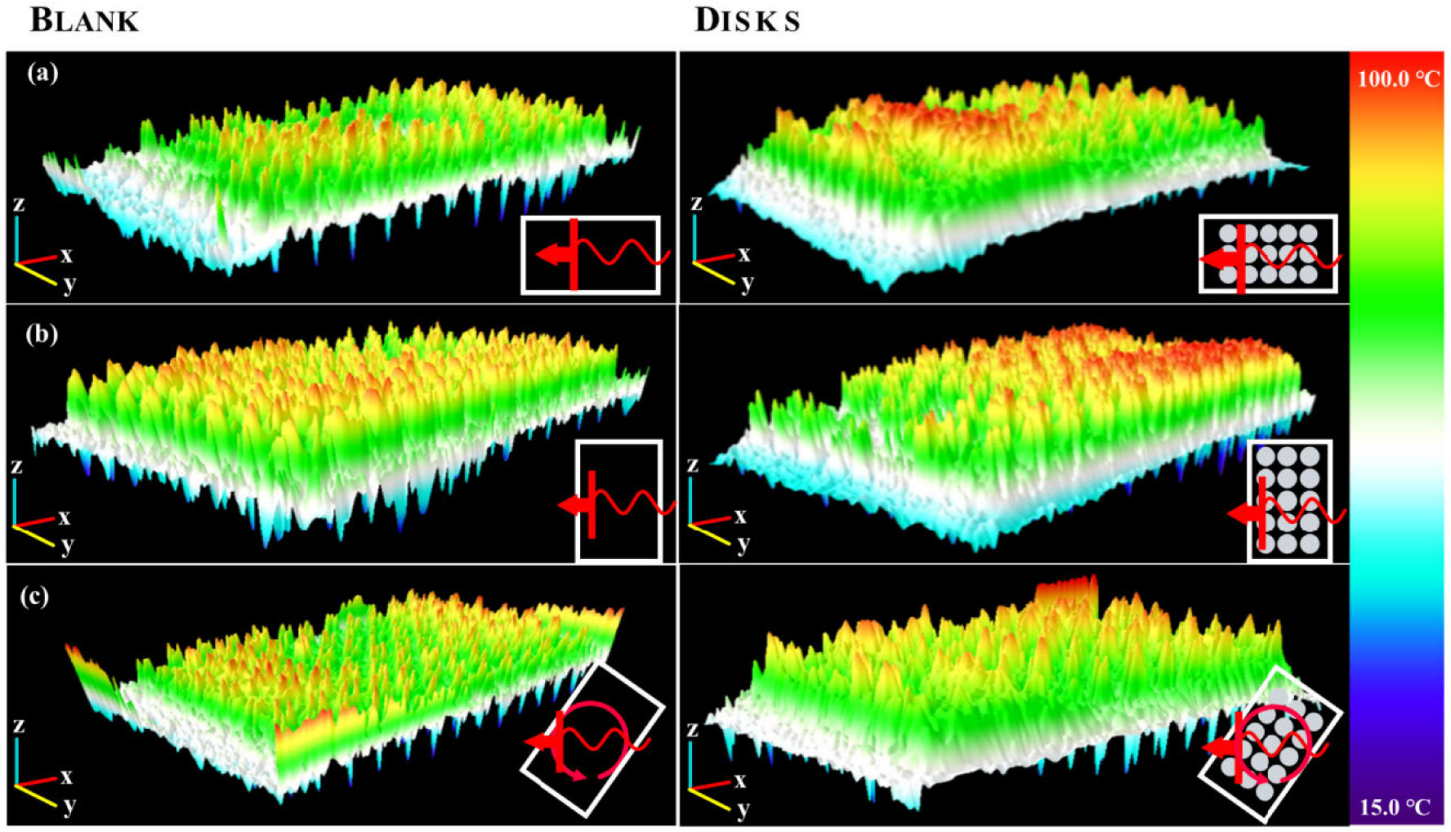
3D visualizations of heating distributions for (a) horizontal, (b) vertical, and (c) rotating orientations of a 96-well microplate with disks (right) compared to a blank 96-well microplate (left) after 30s of heating at 100% power (900 W). See Fig. S7 for array details.

**FIGURE 12.**
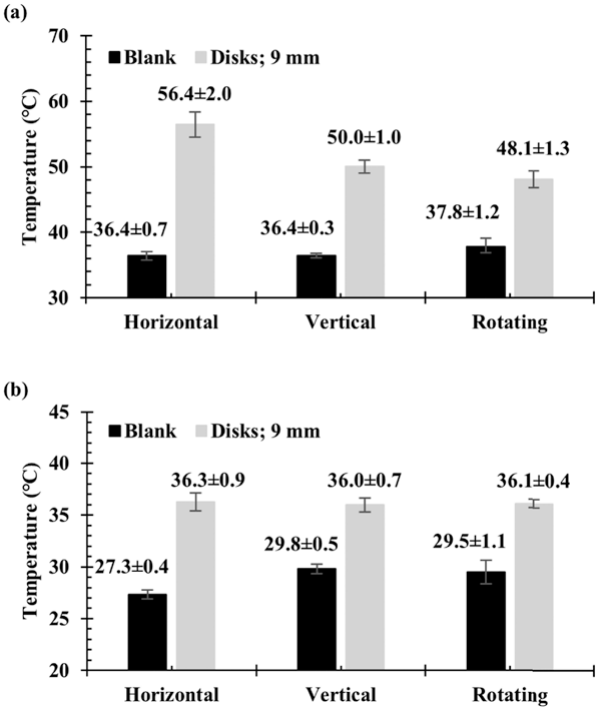
Graphical representation of thermal data from figure 9, comparing (a) the maximum temperature on the microplates with and without the array of disk-shaped PSEs (9 mm radius and 1 mm interelement X and Y spacing) and (b) the average temperature for the microplates with and without array. (n = 5 for each data point).

**FIGURE 13.**
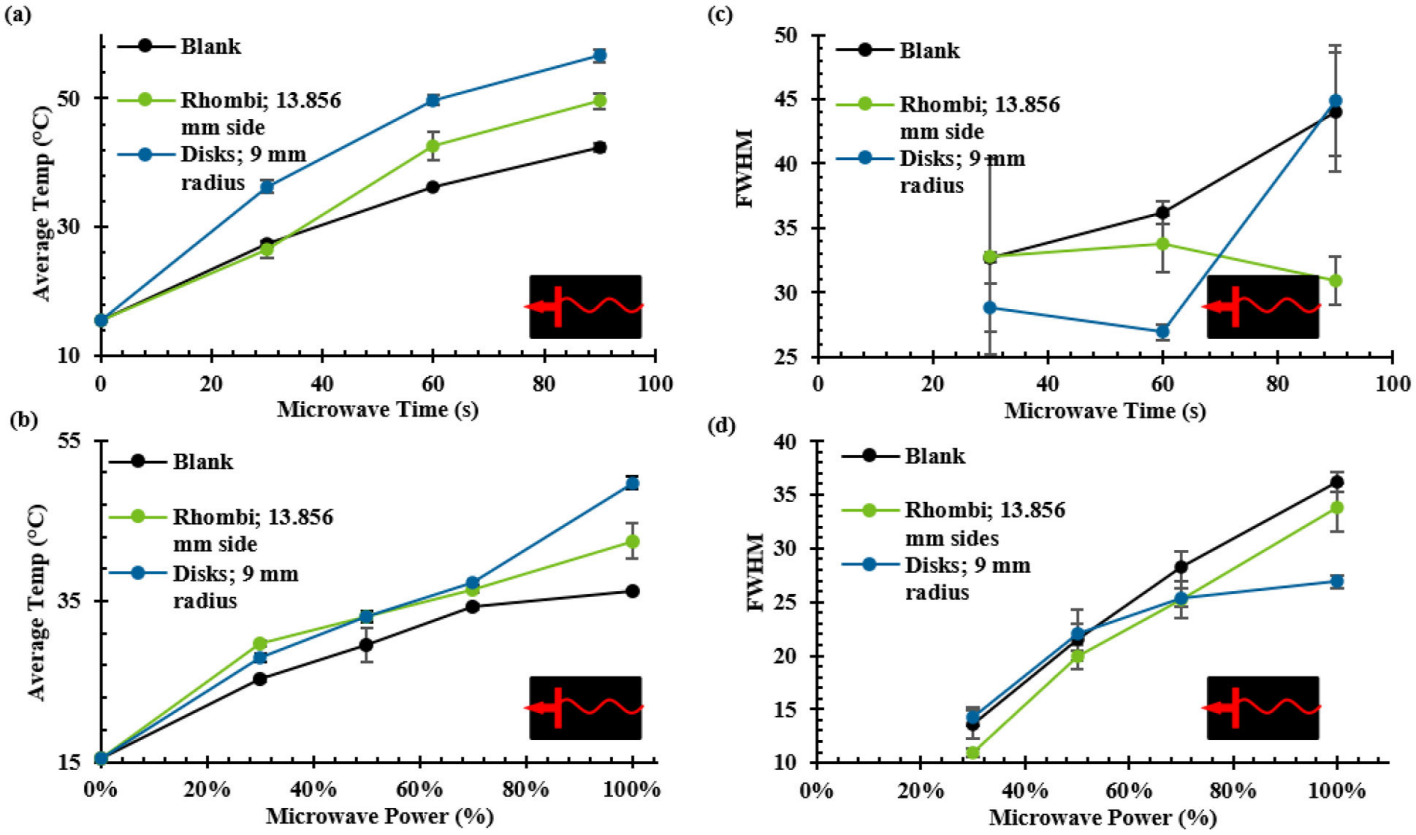
Graphical comparisons of average temperatures and full width at half maximum (FWHM) for (A/C) different microwave times and (B/D) different power levels for horizontally oriented microplates. (n = 5 for each data point).

In thermal experiments, each microplate prototype showed increases in both the average and maximum temperature for all PSE arrays compared to a blank 96-well microplate. While the same four arrays used in the full array FDTD simulations were constructed as prototype microplates, the arrays using 6 mm radius disks and using 20.7846 mm sided rhombi were not fully explored. This was due to the minimal heating effects of the 6 mm radius disk array and the 20.7846 mm sided rhombi array exhibiting destructive tendencies such as arcing, sparking, and microplate combustion in all orientations and power settings. When compared to the E-field contour plots from FDTD results, the distribution profiles appeared to show an inverse correlation between electric field intensity and temperature, as seen in Fig. 9. The FDTD electric field plots and the thermal images of the analogous microplate showed maximum values in the center of the plate for E-field intensity, shown in Fig. 9(a), and just to the left and right of center for temperature, shown in Fig. 9(b). Regular microplates were also shown to have similar heating patterns both with and without deionized water in the wells but exhibited a significant increase in temperature when an array was added to the plate, shown in Fig. 9(b). Different orientations appeared to show results complementary to those observed with the FDTD simulations, with vertically orientated microplates showing greater average and maximum temperatures than their horizontally oriented analogues (See Table 1 in Supplementary Information) but weaker electric field intensities. This relationship was also be observed with other orientations for both the FDTD and thermographic results as shown in Fig. 10, with the greatest electric field intensity in the center of the FDTD contour plot and the thermal images showing greater thermal effects around the edges of the center rather than the center itself. It should be noted that the 55-degree rotated FDTD simulation was chosen over 45-degree rotated orientation due to alignment in the simulation setup and is not exactly analogous to the freely rotating thermal experiment due to it being stationary in the simulation. This is one possible reason that the thermal image of the freely rotating microplate in Fig. 10(e) does not show the same inverse relationship as the FDTD counterpart, but still shows greater heating on the periphery of the center rather than the “true” center just as the other orientations do in Fig. 10(d) and Fig. 10(f). Another noticeable difference between the simulations and thermal experiments is that the area of the microplate closest to the RF source, in this case a cavity magnetron, tends to show greater heating than the areas of the microplate further from the RF source. This is likely due to the fact that the actual microwave oven creates the standing waves used for heating by reflecting waves off the wall opposite to the magnetron to create interference with those leaving the magnetron, meaning that the area of the microplate closer to the magnetron will absorb slightly more irradiation. By comparison, a standing wave was created in the FDTD simulations using two plane wave sources directed at each other to create interference. This implies that the position of the microplate relative to the RF source has a stronger effect on heating than the presence of the PSE array alone does, despite the microplates with PSE arrays showing significantly higher temperatures in all cases. In terms of enhanced heating, the arrays with disk-shaped PSEs shown in Fig. 10(d)-(f) showed considerably greater temperature increases than a regular microplate did under the same conditions, which can be seen in 3D in Fig. 11 and graphically in Fig. 12. The differences in maximum temperature between a regular microplate and one with an array were observed to have differed by as much as 23 °C and the differences in average temperature by as much as 10 °C for each orientation (See Supplementary Information). Overall, microplates in the horizontal orientation appeared to show the highest temperatures for arrays with disk-shaped PSEs though the average temperature is roughly the same regardless of orientation; with the average temperatures being 36.3 ± 0.9 °C for horizontal irradiation, 36.0 ± 0.7 °C for vertical irradiation, and 36.1 ± 0.4 °C for rotation during irradiation for 30 s at 900 W of power. Based on the distributions of the thermal images obtained from these experiments, microplates with the PSE arrays showed lower full width at half-max (FWHM) values and therefore have a smaller distribution of temperatures compared to a blank microplate. When comparing arrays of disk-shaped PSEs and arrays of rhombi-shaped PSEs, the former appears to have a slightly broader distribution but with a smaller peak corresponding to lower temperatures emerging while the latter have lower values for temperature but a narrower distribution, shown in Fig. S8. This appears to suggest that microplates that exhibit higher average temperatures will have a narrower distribution but that at longer heating times this distribution may broaden due to the emergence of peaks representing lower temperatures. Similar analysis of the effects of orientation on the distributions of temperatures show that rotating microplates have a narrower distribution of higher temperatures compared to the microplates in the horizontal and vertical orientations indicating more homogenous heating, as shown in Fig. S9. Compared to the microplate with an array of rhombi-shaped PSEs, the microplate with an array of disk-shaped PSEs shows greater average temperatures at all times and powers of irradiation except for at 270 W of power for 60 s where the temperature of the array of rhombi-shaped PSEs was greater, which can be seen in Fig. 13(a) and (b). In addition, the heating distributions of each array during time-varying experiments appeared to be narrower than those of a blank microplate when compared in terms of full width at half-max (FWHM), as shown in Fig. 13(c), though the deviation of the measurements overlaps to a high degree. In power-varying experiments however, the rhombi array appeared to have a significantly lower FWHM at 30% power (270 W) compared to the disk array and blank microplate, shown in Fig. 13(d).

Data for the vertical and horizontal orientations of each array were also obtained; however, arrays with rhombi-shaped PSEs were found to be highly incompatible with rotation during irradiation due to the previously mentioned destructive tendencies. Arrays with disk-shaped PSEs were found to behave similarly for both rotating irradiation and the vertical orientation at irradiation times longer than 30 s. Despite these difficulties, vertically oriented irradiation suggests the same trends that horizontal irradiation does, shown in Fig. S10. One exception are the arrays of rhombi-shaped PSEs which exhibit greater temperatures than disk arrays at 70% power (630 W) irradiation for 60 s. The distributions of temperatures for each array in the vertical and horizontal irradiation orientations, shown in Fig. S11 and Fig. S12, suggest that FWHM may be similar for the same orientations regardless of whether any array is present. Different orientations of the same array may also exhibit different distributions of temperatures, as exemplified by the microplate with a disk array showing the broadest distribution and lowest peak in the vertical orientation but the narrowest distribution and highest peak in the horizontal orientation. The average temperature and FWHM data for each array in every orientation, time, and power can be seen in Table 1.

## IV. CONCLUSION

As shown in this work, we have developed a viable method of designing a microplate platform for high-throughput sample preparation and two prototype microplates based on the existing MLT platform. Building off of MLTs, we have been able to use FDTD simulations to model our microplate systems *in-silico* before creating prototypes with standard microplates and aluminum foil to observe their thermal properties. From this we have concluded that larger PSEs show greater enhancement of electric field intensity and significant increases in heating during irradiation. In addition to this, microplates in the horizontal orientation show greater electric field enhancements but lower thermal enhancements than microplates in the vertical orientation. Similarly, PSE arrays that showed greater electric field intensity in FDTD simulations, such as 13.856 mm side rhombi-shaped PSEs, tended to exhibit lower temperatures after irradiation than PSE arrays that showed weaker electric field intensity in FDTD simulations, such as 9 mm disk-shaped PSEs. While it has not yet been determined how well these microplate prototypes can process biological samples compared to MLTs, they do show electromagnetic and thermal effects comparable to MLTs them in terms of both timescale and magnitude. Future endeavors will study how well the two rhombi and disk array microplates work for sample preparation by observing the ability of each of them to lyse bacteria, fragment DNA, inactivate enzymes, and the other effects that have been observed with individual MLTs.

## Supplementary Material

supp1-3063092
