# Supplementary material for: Development of a Microplate Platform for High-Throughput Sample Preparation Based on Microwave Metasurfaces
Source: IEEE Access. Author manuscript; Available in PMC 2021 May 13. (PMC8117924; doi:10.1109/access.2021.3063092)
Supplement: supp1-3063092 [file NIHMS1682202-supplement-supp1-3063092.pdf]

SUPPLEMENTARY INFORMATION

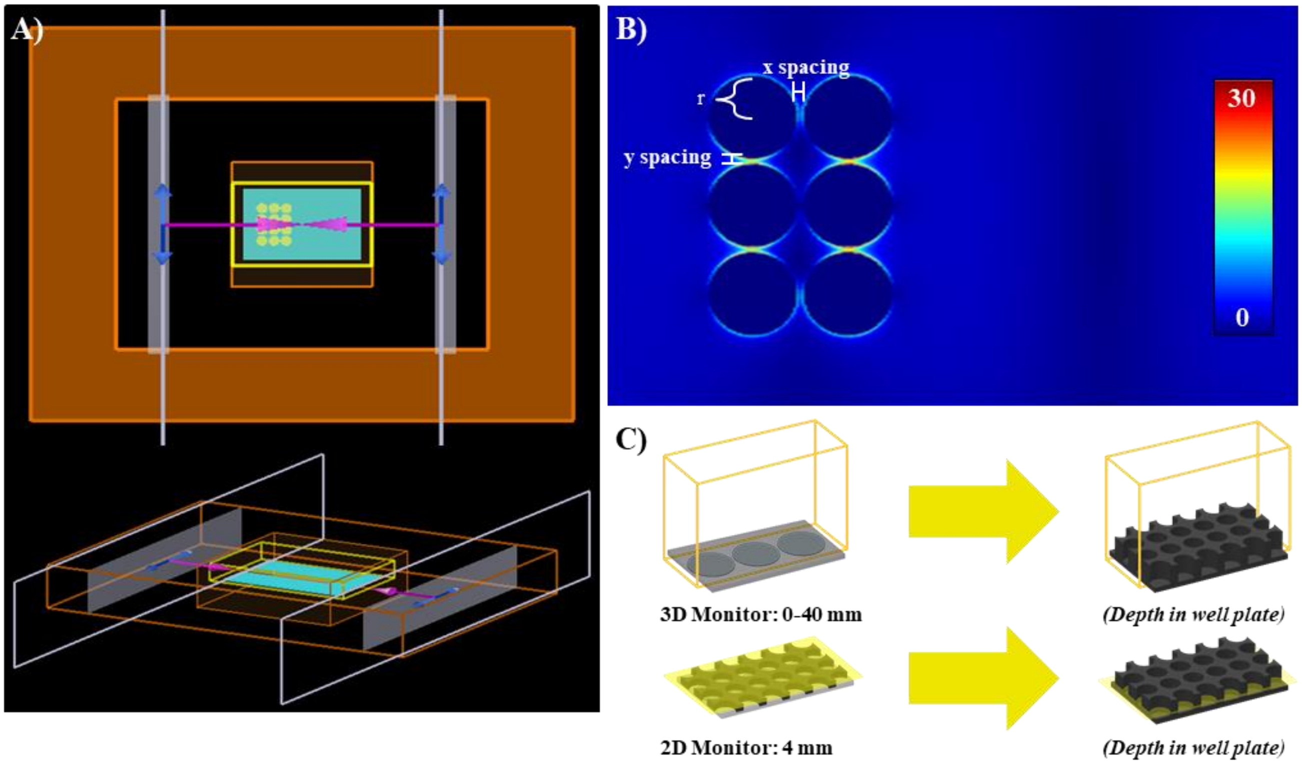

FIGURE S1. **FDTD simulation setup and results: (A) Bottom-up (top) and perspective (bottom) view showing plane wave sources, polystyrene substrate, and array. (B) 2D contour plot showing element spacings and electric field amplitude above the substrate and array for a half-covered simulation. (C) Diagram showing monitor heights in the simulation and where their analogous location on a microplate would be.**

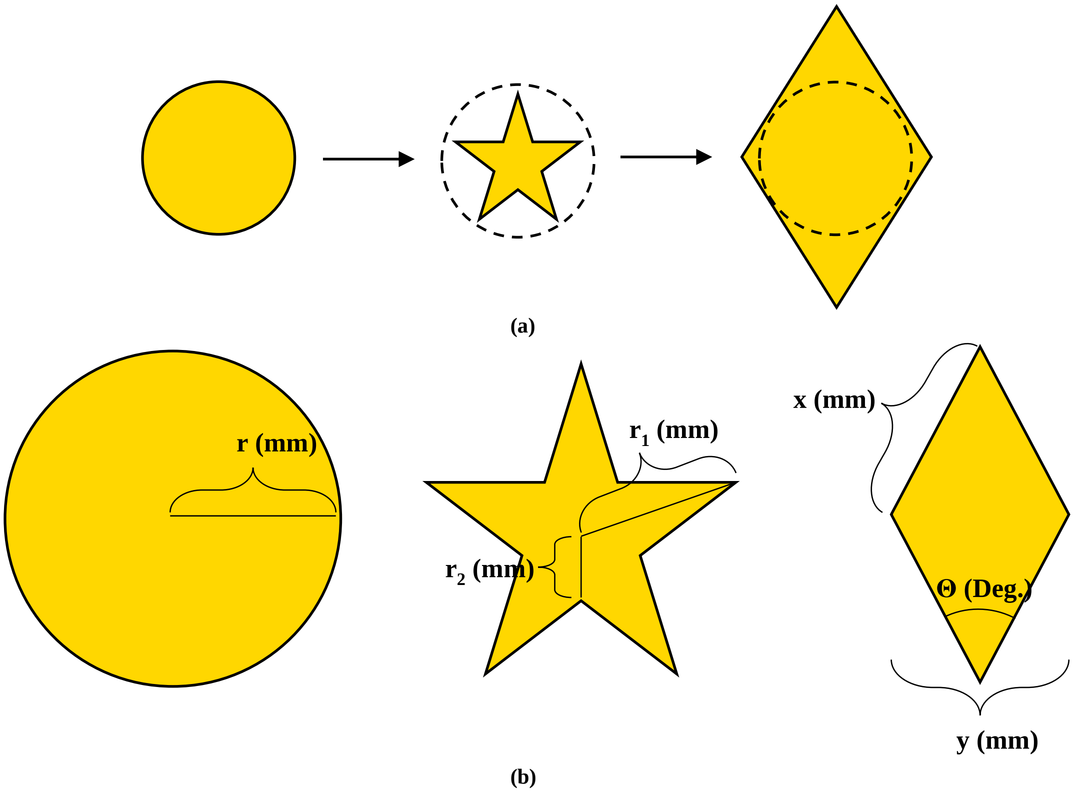

FIGURE S2. (a) Relative array element sizes and the reasoning by which different element shapes and sizes were chosen. (b) Descriptions of element sizes and dimension labels for disks, rhombi, and stars.

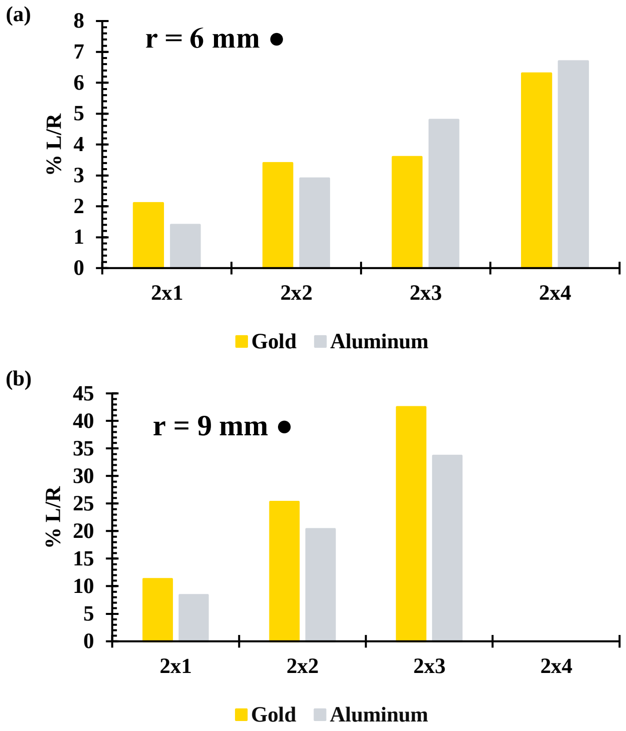

FIGURE S3. **Graphs showing comparisons of %(L/R) between half plate simulations using gold and aluminum array elements for (a) 6 mm radius disks with 3 mm X spacing and 1 mm Y interelement spacing and (b) 9 mm radius disks with 1 mm x and y interelement spacing.**

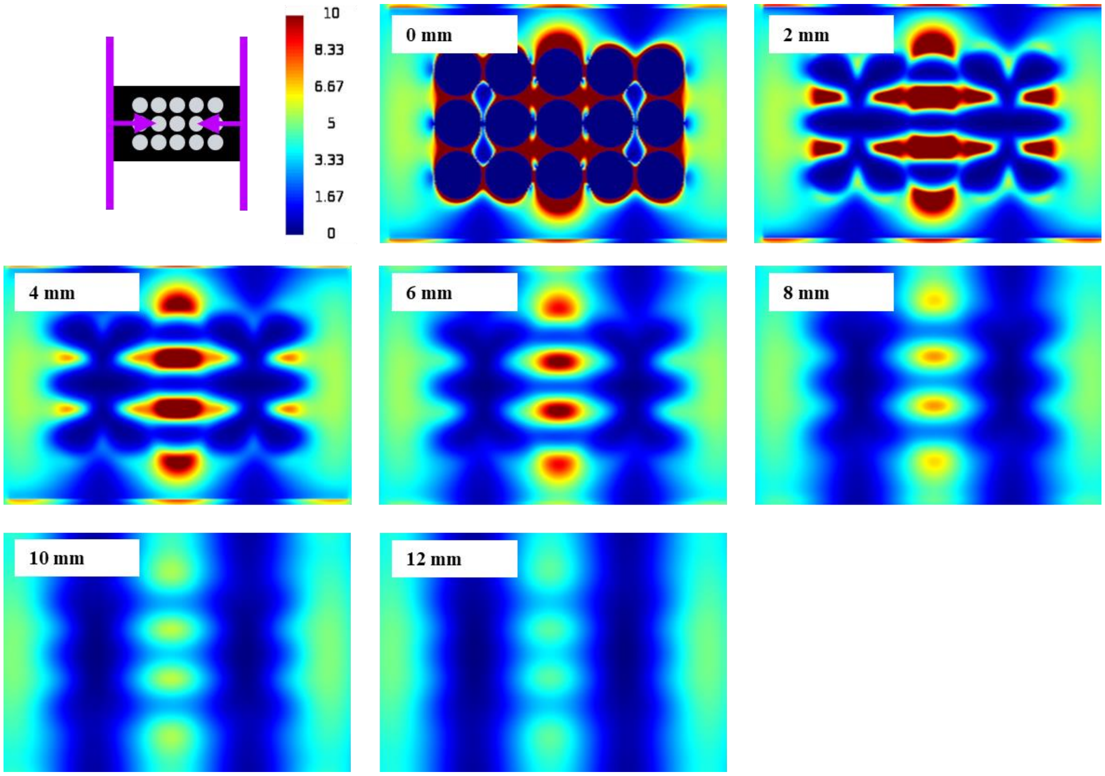

FIGURE S4. **FDTD results showing electric field intensity ($|E|^2$) profiles for disk arrays in the horizontal orientation; 0 mm – 12 mm above array. Monitors were placed at different heights to show how the electric field intensity (V/m) changes with distance from the arrays. The simulations were carried out using two plane wave sources (purple) at 2.45 GHz and 0 Hz bandwidth for one pulse, a polystyrene substrate 12.7 cm by 8.6 cm, and an array of Al disks of radius 9 mm and 1 mm of interelement spacing in the X and Y directions.**

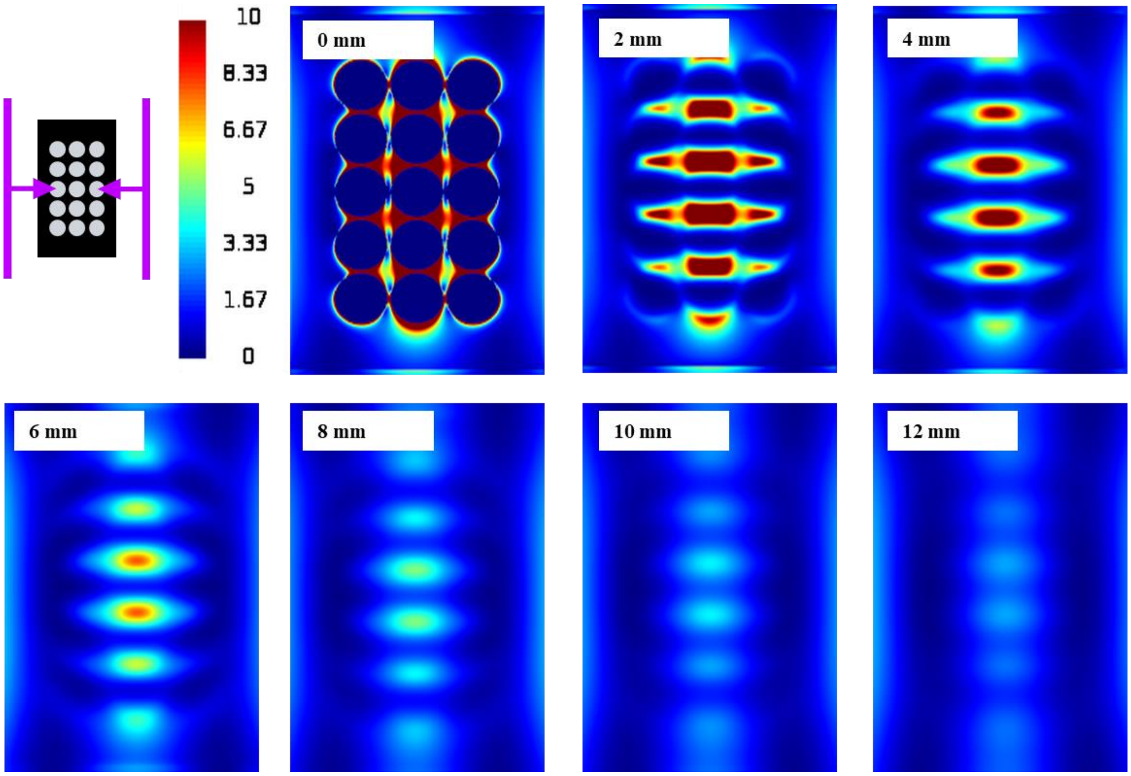

FIGURE S5. **FDTD results showing electric field intensity (|E|²) profiles for disk arrays in the vertical orientation; 0 mm – 12 mm above the array. Monitors were placed at different heights to show how the electric field intensity (V/m) changes with distance from the array. The simulations were carried out using two plane wave sources (purple) at 2.45 GHz and 0 Hz bandwidth for one pulse, a polystyrene substrate 12.7 cm by 8.6 cm, and an array of Al disks of radius 9 mm and 1 mm of interelement spacing in the X and Y directions.**

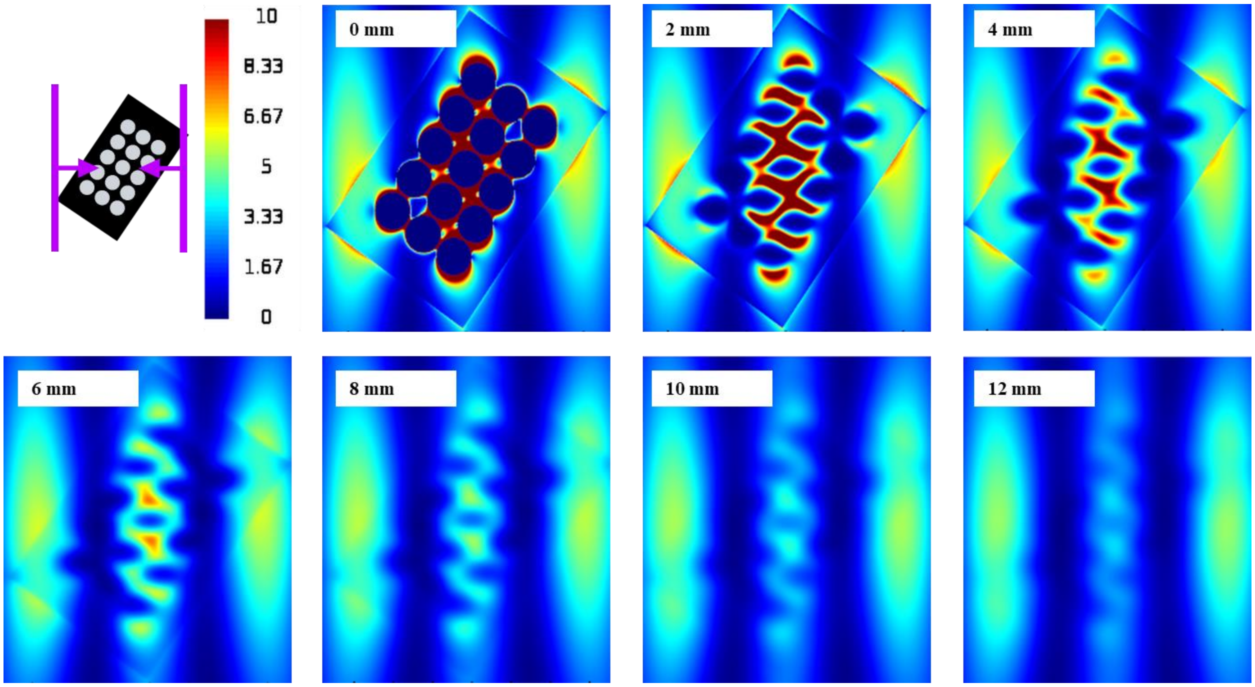

FIGURE S6. **FDTD results showing electric field intensity (|E|^2) profiles for disk arrays in the rotated orientation; 0 mm – 12 mm above array. Monitors were placed at different heights to show how the electric field intensity (V/m) changes with distance from the array. The simulations were carried out using two plane wave sources (purple) at 2.45 GHz and 0 Hz bandwidth for one pulse, a polystyrene substrate 12.7 cm by 8.6 cm, and an array of Al disks of radius 9 mm and 1 mm of interelement spacing in the X and Y directions.**

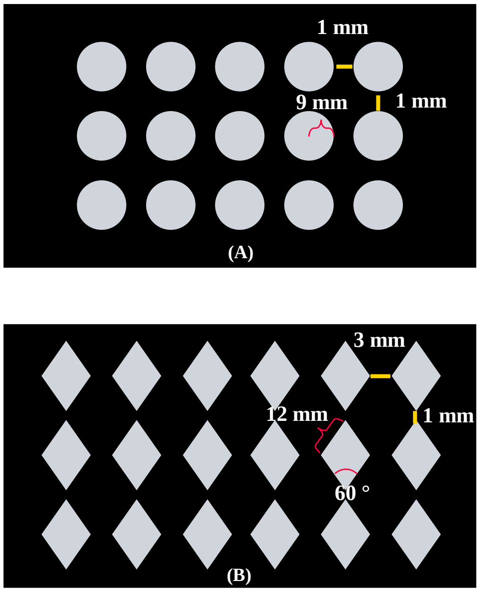

FIGURE S7. **Array dimensions (red) and interelement spacings (yellow) for the (A) disk and (B) rhombi arrays on microplates used for thermal data.**

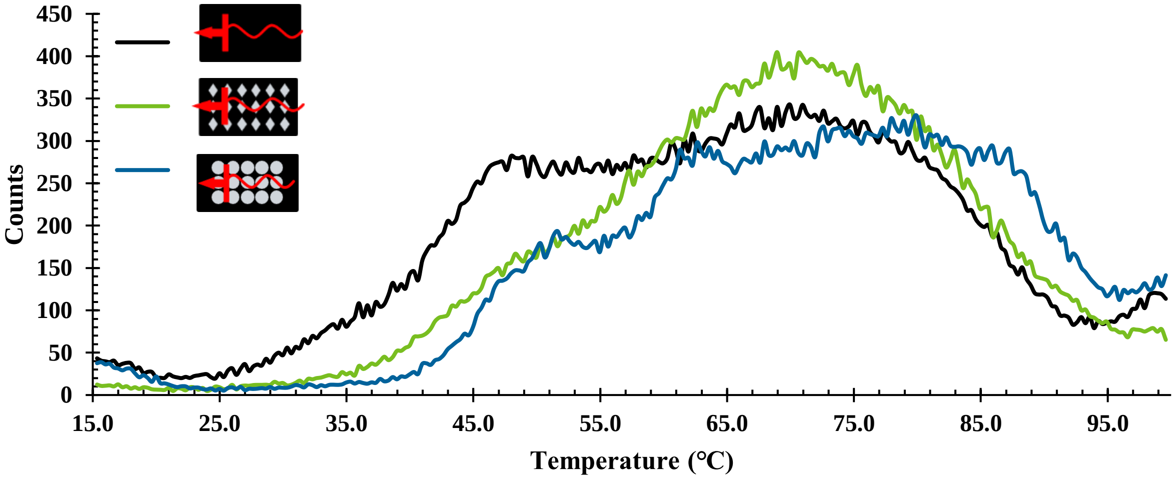

FIGURE S8. Plots of heating distributions for disk and rhombi arrays and a blank microplate after irradiation in the horizontal orientation for 90s at 100% power. Thermal images were converted to greyscale with a temperature range of 15.0 ℃ - 100.0 ℃ and then analyzed using ImageJ to create a distribution of the greyscale pixel values (0-255) corresponding to different temperatures. Note: These values were not used to calculate average or maximum temperature, only to show the shapes and dispersity of the distributions.

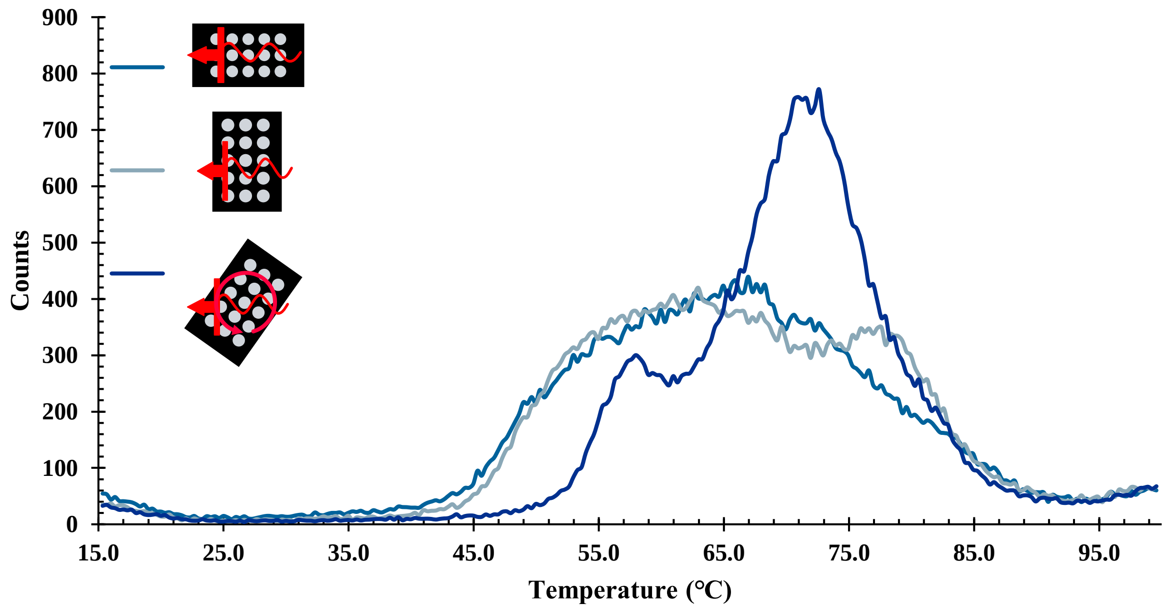

FIGURE S9. Plots showing the breadth of heating distributions for different orientations; horizontal, vertical, and rotating at 30s; 100% power. *Note the temperature values were obtained by converting a greyscale image with a given temperature range (15.0 ℃ - 100.0 ℃) to a range of pixel values (0-255) to obtain the scatter plot. These values were not used to calculate average or maximum temperature, only the shapes and dispersity of the distributions. (n = 5 for each data point).

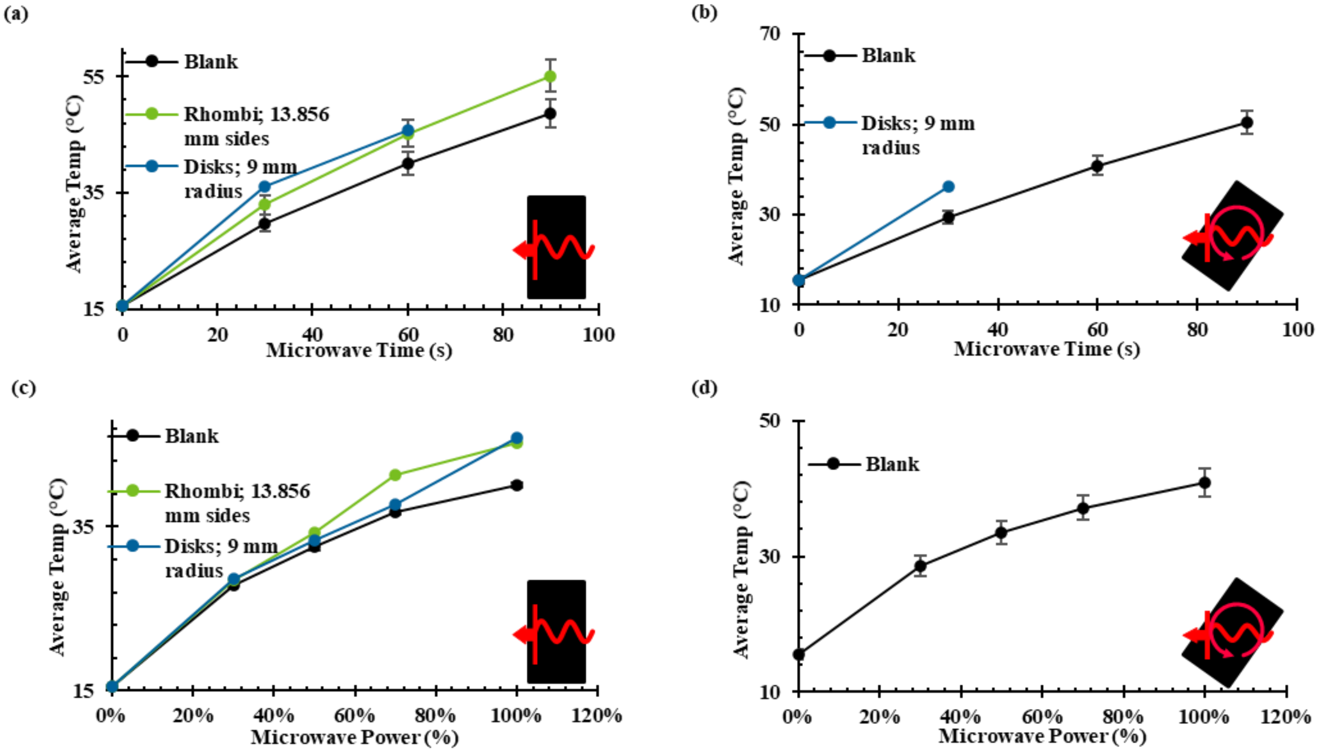

FIGURE S10. Average temperature data for vertical and rotating orientations of blank, rhombi, and disk array microplates for both (a/b) time varying and (c/d) power varying experiments. (n = 5 for each data point) *Note: missing data points were unable to be collected due to destructive tendencies.

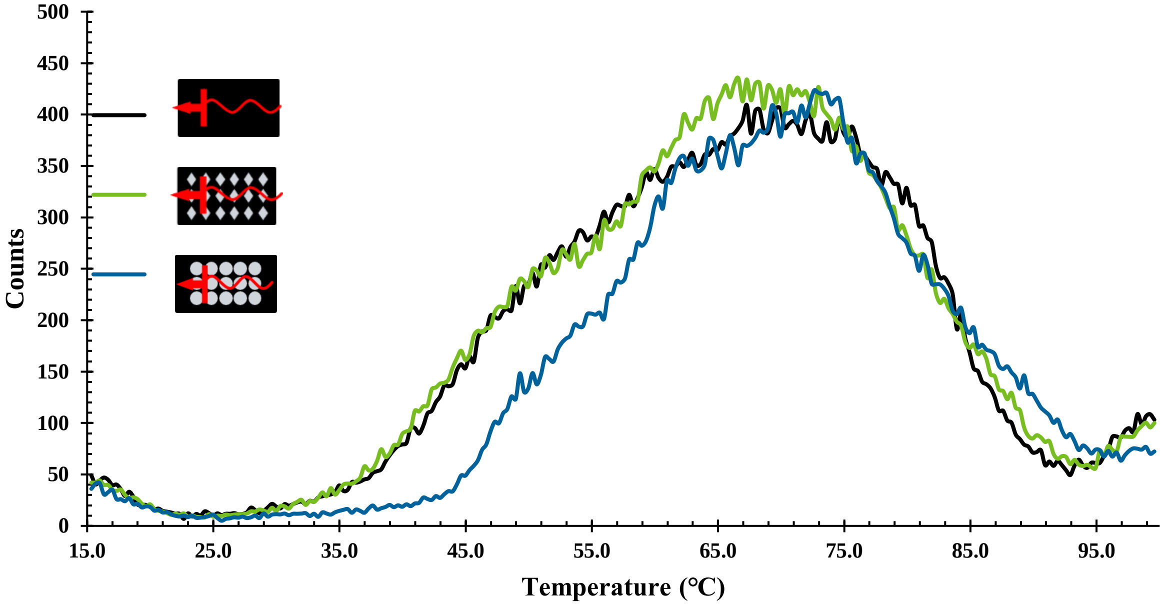

FIGURE S11. **Plot of thermal data for horizontally oriented blank, disk arrayed, and rhombi array microplates after 60 s of irradiation at 100% power (900 W). Thermal images were collected with a forward looking infrared (FLIR) camera and analyzed using FLIR tools software to determine the maximum and average temperatures. These images were then converted into 8-bit greyscale images and converted into histograms using ImageJ to observe the dispersity of each distribution. (n = 5 for each data point).**

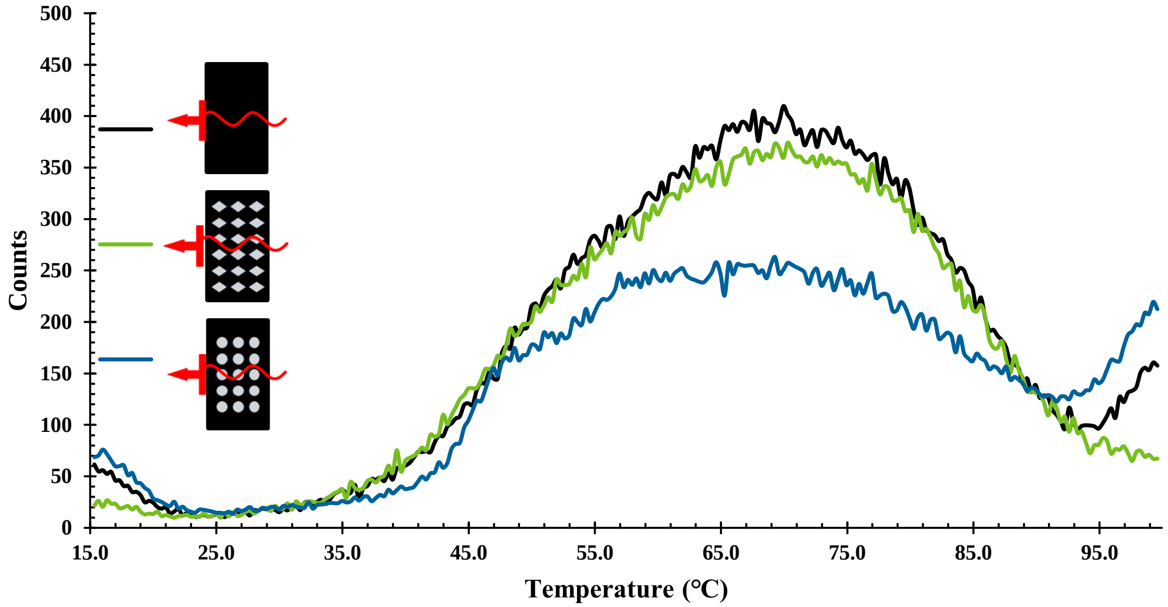

FIGURE S12. **Plot of thermal data for vertically oriented blank, disk arrayed, and rhombi array microplates after 60 s of irradiation at 100% power (900 W). Thermal images were collected with a forward looking infrared (FLIR) camera and analyzed using FLIR tools software to determine the maximum and average temperatures. These images were then converted into 8-bit greyscale images and converted into histograms using ImageJ to observe the dispersity of each distribution. (n = 5 for each data point).**

TABLE I

Average Temperatures and Distribution for
Blank, Disk, and Rhombi Arrays

Array[a,b]	Average Temp (°C)	FWHM (°C)	MW Time (s)	MW Power (W)	Array[a,b]	Average Temp (°C)	FWHM (°C)	MW Time (s)	MW Power (W)
Blank Plate ↔	27.3 ± 0.4	32.7±0.4	30	900	Blank Plate ↔	25.3 ± 0.3	13.6±1.3	60	270
Blank Plate ↕	29.8 ± 0.5	29.8±0.7	30	900	Blank Plate ↕	27.8 ± 0.3	13.0±0.0	60	270
Blank Plate ↻	29.5 ± 1.1	24.7±1.4	30	900	Blank Plate ↻	28.6± 0.6	16.3±0.6	60	270
Disks Plate ↔	36.3 ± 0.9	28.8±1.9	30	900	Disks Plate ↔	28.0 ± 0.5	14.3±0.9	60	270
Disks Plate ↕	36.0 ± 0.7	31.8±1.0	30	900	Disks Plate ↕	28.6 ± 0.2	20.0±0.9	60	270
Disk Plate ↻	36.1 ± 0.4	11.8±0.6	30	900	Disk Plate ↻	No Data	No Data	60	270
Rhombi Plate ↔	26.5± 1.3	32.8±7.6	30	900	Rhombi Plate↔	29.7± 0.3	10.9±0.4	60	270
Rhombi Plate ↕	33.0± 0.2	32.6±1.1	30	900	Rhombi Plate ↕	28.5± 0.7	20.3±1.2	60	270
Rhombi Plate ↻	No Data	No Data	30	900	Rhombi Plate ↻	No Data	No Data	60	270
Blank Plate ↔	36.3 ± 0.1	36.2±0.9	60	900	Blank Plate ↔	29.6 ± 2.1	21.5±2.8	60	450
Blank Plate ↕	40.0 ± 0.3	40.8±6.4	60	900	Blank Plate ↕	32.5 ± 0.4	20.0±1.4	60	450
Blank Plate ↻	40.9± 0.8	33.2±1.8	60	900	Blank Plate ↻	33.5± 0.5	24.1±1.1	60	450
Disks Plate ↔	49.7 ± 0.8	26.9±0.6	60	900	Disks Plate ↔	33.1 ± 0.7	22.0±1.0	60	450
Disks Plate ↕	45.8 ± 0.6	52.9±1.0	60	900	Disks Plate ↕	33.4 ± 0.4	22.6±0.4	60	450
Disk Plate ↻	No Data	No Data	60	900	Disk Plate ↻	No Data	No Data	60	450
Rhombi Plate↔	42.5± 2.2	33.8±2.2	60	900	Rhombi Plate↔	33.0± 0.3	20.0±0.5	60	450
Rhombi Plate ↕	45.2± 2.0	39.2±4.9	60	900	Rhombi Plate ↕	34.3± 0.6	29.1±1.0	60	450
Rhombi Plate ↻	No Data	No Data	60	900	Rhombi Plate ↻	No Data	No Data	60	450
Blank Plate ↔	42.4 ± 0.6	44.0±4.6	90	900	Blank Plate ↔	34.3 ± 0.3	28.3±1.4	60	630
Blank Plate ↕	48.6 ± 0.8	40.5±1.3	90	900	Blank Plate ↕	36.7 ± 0.3	27.1±0.8	60	630
Blank Plate ↻	50.4± 0.7	36.7±1.6	90	900	Blank Plate ↻	37.2± 0.3	29.6±1.8	60	630
Disks Plate ↔	56.6 ± 0.9	44.9±4.3	90	900	Disks Plate ↔	37.3 ± 0.3	25.4±0.9	60	630
Disks Plate ↕	No Data	No Data	90	900	Disks Plate ↕	37.7 ± 0.4	24.5±1.8	60	630
Disk Plate ↻	No Data	No Data	90	900	Disk Plate ↻	No Data	No Data	60	630
Rhombi Plate↔	49.6± 1.2	30.9±1.9	90	900	Rhombi Plate↔	36.5± 0.4	25.2±1.7	60	630
Rhombi Plate ↕	55.1± 1.8	38.2±2.9	90	900	Rhombi Plate ↕	41.3± 1.0	34.8±2.4	60	630
Rhombi Plate ↻	No Data	No Data	90	900	Rhombi Plate ↻	No Data	No Data	60	630

[a]See supplemental Fig. S7 for array details.

[b]Annotation: ↕ = vertical, ↔ = horizontal, ↻ = rotating orientations

[c]See supplemental Fig. S11 and S12 for distribution histograms.

"MW" = microwave

"FWHM" = full width at half maximum
